# Local adaptation through genetic differentiation in highly fragmented *Tilia cordata* populations

**DOI:** 10.1002/ece3.4131

**Published:** 2018-05-07

**Authors:** Albin Lobo, Ole Kim Hansen, Jon Kehlet Hansen, Eva Ortvald Erichsen, Birgitte Jacobsen, Erik Dahl Kjær

**Affiliations:** ^1^ Department of Geosciences and Natural Resource Management (IGN) University of Copenhagen Frederiksberg C Denmark; ^2^ Present address: Ministry of Fisheries and Hunting Nuuk Greenland

**Keywords:** drift, *F*_ST_, gene flow, population genetics, *Q*_ST_, selection

## Abstract

We assessed the level of geographic differentiation of *Tilia cordata* in Denmark based on tests of 91 trees selected from 12 isolated populations. We used quantitative analysis of spring phenology and population genetic analysis based on SSR markers to infer the likely historical genetic processes within and among populations. High genetic variation within and among populations was observed in spring phenology, which correlated with spring temperatures at the origin of the tested *T. cordata* trees. The population genetic analysis revealed significant differentiation among the populations, but with no clear sign of isolation by distance. We infer the findings as indications of ongoing fine scale selection in favor of local growth conditions made possible by limited gene flow among the small and fragmented populations. This hypothesis fits well with reports of limited fruiting in the investigated Danish *T. cordata* populations, while the species is known for its ability to propagate vegetatively by root suckers. Our results suggest that both divergent selection and genetic drift may have played important roles in forming the genetic patterns of *T. cordata* at its northern distribution limit. However, we also speculate that epigenetic mechanism arising from the original population environment could have created similar patterns in regulating the spring phenology.

## 1 INTRODUCTION

### 1.1 Natural selection vs. neutral genetic processes in local adaptation

The potential for a species to locally adapt to particular climatic and soil conditions is assumed to depend on the genetic variation within and among its individual populations (Aitken, Yeaman, Holliday, Wang, & Curtis‐McLane, 2008; Kawecki & Ebert, 2004), which in turn is dependent on various factors such as species’ life‐history traits and genetic processes in the landscape (Franks, Weber, & Aitken, 2014). Hence, understanding the genetics behind the local adaptation is very important under the expected climate change impact scenario on forest ecosystems (Savolainen, Lascoux, & Merilä, 2013). Phenotypic plasticity in adaptive traits aids fast adaptation (Valladares et al., 2014), but plasticity will probably not be enough for tree species to adapt to the ongoing change in climate (Duputié, Rutschmann, Ronce, & Chuine, 2015). Rather, high levels of genetic variation within populations and among populations are probably required for continuous adaptation to a changing climate (Aitken & Whitlock, 2013; Franks et al., 2014). Among population variation is especially important, as it introduces new alleles through gene flow (Kremer et al., 2012). Selection and random drift occur simultaneously in natural populations and have in the case of tree species often resulted in the occurrence of genetically differentiated populations over large geographic ranges (Alberto et al., 2013; Nadeau, Meirmans, Aitken, Ritland, & Isabel, 2016). The balance between neutral processes and natural selection is important, and the ability of a species to adapt to particular set of local growth conditions can be limited if neutral processes dominate the evolution of among population variation (Savolainen, Pyhäjärvi, & Knürr, 2007).

### 1.2 Genetic differentiation in fragmented landscapes

Habitat fragmentation can have a negative influence on a species’ capacity to adapt to new conditions by reducing genetic diversity and increasing inbreeding in small populations (Lowe, Cavers, Boshier, Breed, & Hollingsworth, 2016). However, tree species in fragmented landscapes often maintain connectivity through extensive gene flow in the form of pollen movement and seed dispersal (Breed, Ottewell, Gardner, & Lowe, 2011). On the other hand, a reduced gene flow among populations can be beneficial for local adaptation, because site‐specific fitness of adaptive alleles may only lead to local adaptation in the absence of homogenizing gene flow (Sork, 2016) *. *Trees are long‐lived organisms, and it is therefore difficult to estimate effects of divergent selection directly by comparing development over several generations. Instead, phenotyping in common garden trials or provenance field trials are applied to compare the performance of trees that originate from different climatic conditions. In such studies, evidence of natural selection is inferred from the level and pattern of genetic differentiation among origins from divergent climatic conditions (Morgenstern, 1996). More recently, landscape genomics involving correlation of allele frequencies and environmental conditions has been applied to infer effects of selection, although the efficiency of the approach with the present level of genomic data is still discussed (Ćalić, Bussotti, Martínez‐García, & Neale, 2015). Estimation of gene flow across landscapes based on genetic markers is, however, well established, and results from a large number of studies of trees are available (Lowe, Cavers, Boshier, Breed, & Hollingsworth, 2015; Savolainen et al., 2007). A combination of the different approaches is therefore ideal for studying the genetic background of local adaptation of trees in fragmented landscapes (Lepais & Bacles, 2014; Sork et al., 2013).

*Tilia cordata* Mill. (small‐leaved lime) is native to Europe with its Northern distribution limit at the Southern part of Finland, Sweden, and Norway. Being close to its Northern limits, native Danish *T. cordata* mostly occur in small isolated populations in ancient forests (Lawesson, 2004). The species was dominating in Danish forests until 2,500 years ago (Hannon, Bradshaw, & Emborg, 2000), and the present fragmented distribution of *T. cordata* can therefore be a result of relatively early colonization by the species after postglaciation followed by recurring events of local extinction during the recent part of Holocene, a development that may have taken place in other parts of the natural distribution as well (Fineschi, Salvini, Taurchini, Carnevale, & Vendramin, 2003). Gene flow among the remnant populations is likely to be limited at the northern limit (Myking, 2002), especially due to limited production of fertile seeds (Pigott & Huntley, 1981), which is also observed in many of the Danish populations included in this study (Lawesson, 2004). While the pronounced ability of *T. cordata* to reproduce through root and stump suckers (Koop, 1987a,b) can maintain the population size and genetic diversity within populations, it will result in regeneration without gene flow even among closely located populations.

### 1.3 Objective of the study

In this study, we investigate the hypothesis that divergent selection (which possibly has occurred during thousands of years) combined with very low level of gene flow among the presently very fragmented populations has led to adaptation at a very local scale in Denmark. Also, we study the level of genetic diversity based on the expectation that the life‐history characteristics of the species such as pollination mechanism determine the genetic differentiation happening within and among populations.

In order to separate the effects of genetic drift and selection, we compare the level of population differentiation in the quantitative genetic trait spring phenology (*Q*_ST_ value) with population differentiation based on putative neutral SSR markers (*F*_ST_ value). The presence of divergent natural selection will be indicated by *Q*_ST_ > *F*_ST_ and uniform or stabilizing selection by *Q*_ST_ < *F*_ST_, while *Q*_ST_ = *F*_ST_ suggests that genetic drift is the major driver behind differentiation among populations in the studied traits (Leinonen, Scott McCairns, O'Hara, & Merilä, 2013; Whitlock & Guillaume, 2009). This is because the *Q*_ST_ value denotes the entire population differentiation in a given trait due to combined effect of natural selection and neutral processes, while *F*_ST_ at neutral loci only measures genetic distance between populations arising as a result of neutral processes such as gene flow and drift (De Kort, Vandepitte, & Honnay, 2012). We test if any observed population differentiation in spring phenology can be explained by climatic differences at the locations, or if the patterns mainly reflect geographic distances between populations. We study spring phenology, as there are indications of bud burst advancement in *T. cordata* (Kramer, 1995), and hence to test if the investigated populations are prone to late spring frost damages in the future. Finally we discuss, what can be learned from the findings in relation to the species’ ability to adapt its phenology to the expected changes in the future due to climate change.

## 2 MATERIALS AND METHODS

### 2.1 Plant material

**Figure 1:**
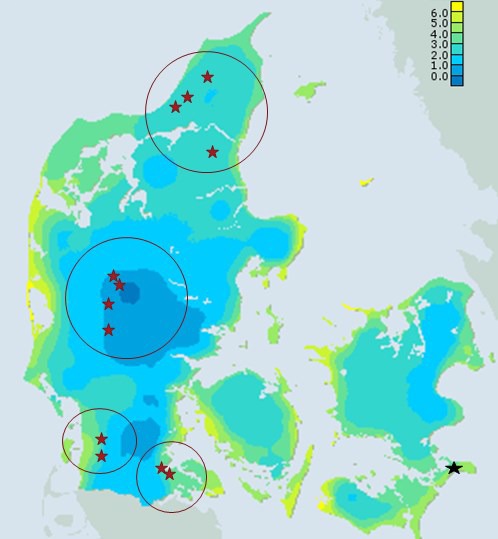
Minimum May temperature and location of the 12 populations (red stars) included in the study (Map provided by Mikael Scharling, Danish Meteorological Institute) and with an outline of four eco‐geographic regions. Phenology was assessed in a clonal seed orchard with replications (black star)

The study was based on 91 trees (clones) selected for the Danish genetic resource conservation program (Graudal, Kjær, & Canger, 1995) from 12 different populations; all of putative native origin from four different eco‐geographic regions in the Western part of Denmark (Figure 1). Scions were collected from the trees in winter 1995/1996, grafted and a clonal seed orchard/test established in Southeast Denmark (55.0076°N, 12.3075°E) in 1998 with single tree plots in six randomized complete blocks, each clone represented once in each block.

### 2.2 Quantitative genetic analysis of spring phenology

Bud burst of all trees in the clonal test was assessed on 19 April 2004 and 5 May 2006 using a scale from 1 to 6 based on the development stage of bud burst from fully closed winter buds (score 1) to fully unfolded leaves (score 6). From these bud burst data, population values (least square mean values [LSMeans]) and variance components between populations and between clones within populations were estimated using model (1) given below:

(1) $$Y_{\mathrm{ijkl}}=\mu +B_{i}+P_{j}+\lambda_{\mathrm{ij}}+C_{k(j)}+\varepsilon_{\mathrm{ijkl}},$$

where *Y*_*ijkl*_ is the bud burst score measured for tree *l*, μ is the overall mean of the bud burst score, *B*_*i*_ is the fixed block effect, *P*_*j*_ is the fixed population effect, λ_*ij*_ is the fixed population by block interaction, *C*_*k(j)*_ is the random effect of clones within population, and ε_*ijkl*_ is the residual. The broad sense heritability (*H* ^2^) was calculated according to Falconer and Mackay (1996) as *V*_G_ */V*_P_ where *V*_G_ is the estimated clonal variance, *V*_P_ is the total phenotypic variance calculated as *V*_G_ + *V*_E_, where *V*_E_ is the estimated residual (environmental) variance in the clonal trial. We estimated the expected response to selection (*R*) for bud burst using breeder's equation following Falconer and Mackay (1996) as follows;

*R = iH* ^2^ *√V*_P*,*_ where *i* is the selection intensity, *H* ^2^ is the broad sense heritability, and *V*_P_ is the phenotypic variance.

We used a scenario based on selection of the 5% and 10% most extreme phenotypes. We compared this measure of expected response from one round of strong selection with the present differences among populations in order to illustrate the magnitude of the present levels of population differentiation in timing of budburst.

The actual level of genetic differentiation in bud burst was calculated following Spitze (1993) as *Q*_ST_ = *V*_POP_/(*V*_POP_ + 2*V*_G_), where *V*_POP_ is the variance between populations and *V*_G_ is the estimated genetic variance of the clones. *Q*_ST_ values are downwards biased as we use the total genetic (clonal) variance as proxy for the additive genetic variance (Goudet & Büchi, 2006; López‐Fanjul, Fernández, & Toro, 2003). The software program ASReml v3.0 was used to estimate variance components and provenance values as well as standard errors of broad sense heritability estimates and *Q*_ST_ estimates (Gilmour et al. 2009).

### 2.3 Molecular analysis based on SSR markers

Nuclear DNA was extracted from the sampled leaves with the QIAGEN^®^ DNeasy 96 Plant Kit (Germany), using approximately 40 mg of leaf material and following the manufactures instructions. The samples were genotyped for nine nuclear microsatellites developed by Phuekvilai and Wolff (2013). Tc23 was not described in Phuekvilai and Wolff (2013), but included in Hansen, Thomsen, and Rasmussen (2014). The microsatellites were amplified in three different multiplex primer mixes using Qiagen multiplex PCR kit (Germany) and following the PCR protocol optimized by Phuekvilai and Wolff (2013). The PCR products were visualized by capillary electrophoresis on an ABI 3130xl sequencer (Applied Biosystems), and size standard GZ500LIZ was applied for reference of fragment size and scored using GeneMapper v.4.0 (Applied Biosystems).

Genetic diversity in the four eco‐geographic regions used in the study was estimated for each locus using the following parameters: observed heterozygosity (*H*_o_), expected heterozygosity (*H*_e_), number of observed alleles (*N*_a_), effective number of alleles (*N*_e_), and allelic richness calculated via rarefaction (*N*_a_[rar]). The first measures were calculated in GenAlEx ver. 6.5 (Peakall & Smouse, 2006, 2012) while allelic richness was calculated in the HP‐Rare 1.1 software (Kalinowski, 2005). Pairwise *F*_ST_ values between clones and between trees pooled in eco‐geographic regions were calculated in GenAlEx 6.502 (Peakall & Smouse, 2005).

### 2.4 Genetic and phenotypic difference among clones as a function of geographic distance

We used a mantel test implemented in the package ade4 in R version 3.2.2 (Dray & Dufour, 2007) to test how pairwise geographic distances among populations correlate with the pairwise differences among populations in timing of bud burst score, and with the pairwise *F*_ST_ values of the clones within and among these populations. The pairwise geographic distance between the populations was calculated using the Geographic Distance Matrix Generator; version 1.2.3 (Ersts, 2011).

### 2.5 Climatic clines in spring phenology

Population bud burst (LSmeans of scores from the clonal test) was correlated against the minimum May temperatures at site of origin in order to test whether the observed patterns indicate climate adaptation to the risk of early spring frost. Population bud burst scores were weighted by the inverse of their variance for increased precision in the procedure REG in SAS (SAS Institute Inc., 2011). Provenance site estimates of average minimum temperature in May were available for the years 1999–2008 from the Danish Meteorological Institute (Scharling, 2017).

## 3 RESULTS

### 3.1 Quantitative genetic variation in spring phenology

**Figure 2:**
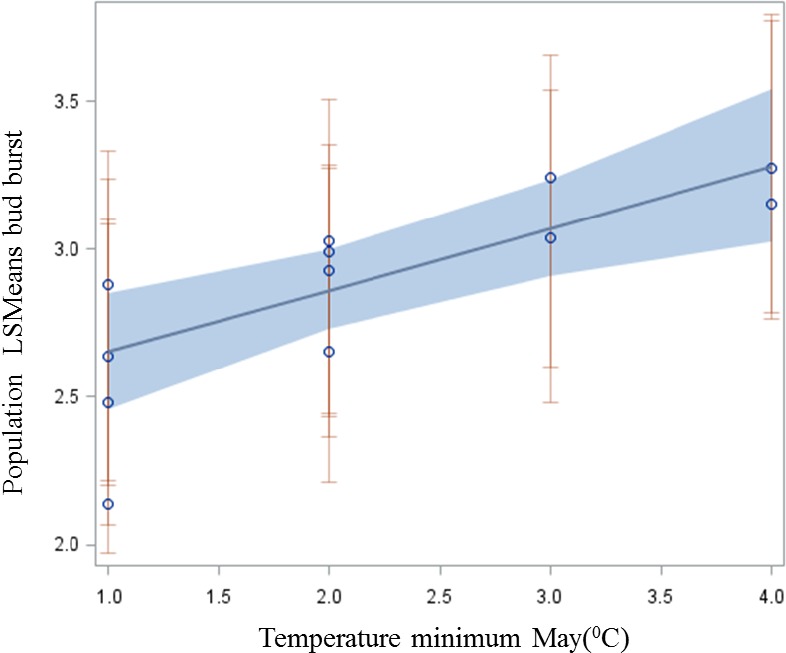
Weighted regression between population means of bud burst score in May 2006 and minimum temperature in May at original population site

**Figure 3:**
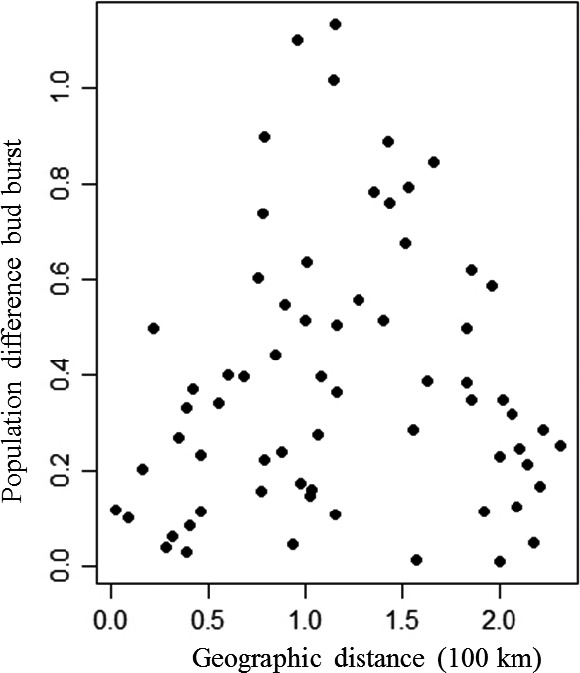
Population differentiation (difference in LSMeans) in spring phenology (=bud burst) plotted against their pairwise geographic distances

The maximum distance among the 12 populations studied was only ~300 kilometers, and the difference in their mean annual temperatures (MAT) was <1°C. The average bud burst score ranged between 1.34 and 2.93 when assessed in April 2004 and between 2.14 and 3.28 when assessed in May 2006 (Table 1). Populations were significantly different as regards bud burst with *Q*_ST_ values 0.25 and 0.33 (Table 2). The expected responses to a selection (of 5% and 10% most extreme individuals for bud burst) were about ½ to ⅓ of the maximum differences found between populations (Table 2). Clones within the populations were significantly different to each other in bud burst (*p* < .001 for all assessments), and the broad sense heritability for bud burst was 0.44 and 0.53 for the two assessments, respectively (Table 2). Population least square means in bud burst showed significant correlation to the average minimum temperature in May (*r* = .75; *p*‐value *= *.004) at the 12 original population sites belonging to the four climatically distinct eco‐geographic regions in Denmark (Figure 2). Pairwise geographic distance between populations and their corresponding differences in LSMeans of bud burst score showed a tendency to be related (*r* = .22; *p*‐value = .06) (Figure 3).

**Table 1:** Climatic data, location, elevation, and average bud burst score of the studied *T. cordata* populations

Population	Latitude (º North)	Longitude (º East)	Altitude (m)	No. of clones	*T*_min_ May (ºC)	Bud burst April 19 2004	Bud burst May 5 2006
Bolderslev skov	55.00	9.36	56.90	8	3	2.82	3.28
Bøgebakke	57.01	9.50	77.30	4	2	2.38	2.93
Draved Skov	55.02	8.97	20.00	9	4	2.75	3.24
Ersted Skov	56.80	9.78	32.80	10	2	1.76	2.66
Holt Krat	56.08	9.45	105.40	10	1	2.05	2.88
Hønning	55.18	8.94	36.10	5	4	2.32	3.04
Kraruplund	55.71	8.65	18.40	10	1	1.83	2.64
Sevel Krat	56.45	8.87	28.00	3	1	1.55	2.48
Skovbjerg Krat	55.93	8.68	19.00	5	1	1.34	2.14
Skovsgårdslund	57.10	9.49	14.10	6	2	2.61	3.03
Åbybjerget	57.19	9.76	11.30	11	2	2.44	2.99
Årslev Skov	55.02	9.37	76.00	10	3	2.93	3.16

**Table 2:** Population differentiation and genetic parameters for spring phenology (*H* ^2^ = Broad sense heritability across populations, *V*_G_ = genetic variance across populations, *V*_P_ = Phenotypic variance, *CV*_G_ = genetic coefficient of variation, Pop Diff = maximum difference between populations, *R*_5_ and *R*_10_ = predicted response to a 5% and 10% selection for bud burst among clones within populations at selection intensities of 2.06 and 1.75, respectively)

Parameters	Bud burst 19 April 2004	Bud burst 5 May 2006
Mean	2.26	2.91
*V*_G_	0.22	0.12
*V*_P_	0.41	0.27
*CV*_G_	0.21	0.12
*H* ^*2*^	0.53	0.44
*SE* (*H* ^*2*^)	0.05	0.06
*Q*_ST_	0.33	0.25
*SE (Q*_ST_ *)*	0.12	0.11
*p*‐value pop	<.001	<.001
Pop Diff	1.6	1.1
*R*_5_ (*i* = 2.06)	0.7	0.5
*R*_10_ (*i* = 1.75)	0.6	0.4

### 3.2 Molecular genetic variation

**Figure 4:**
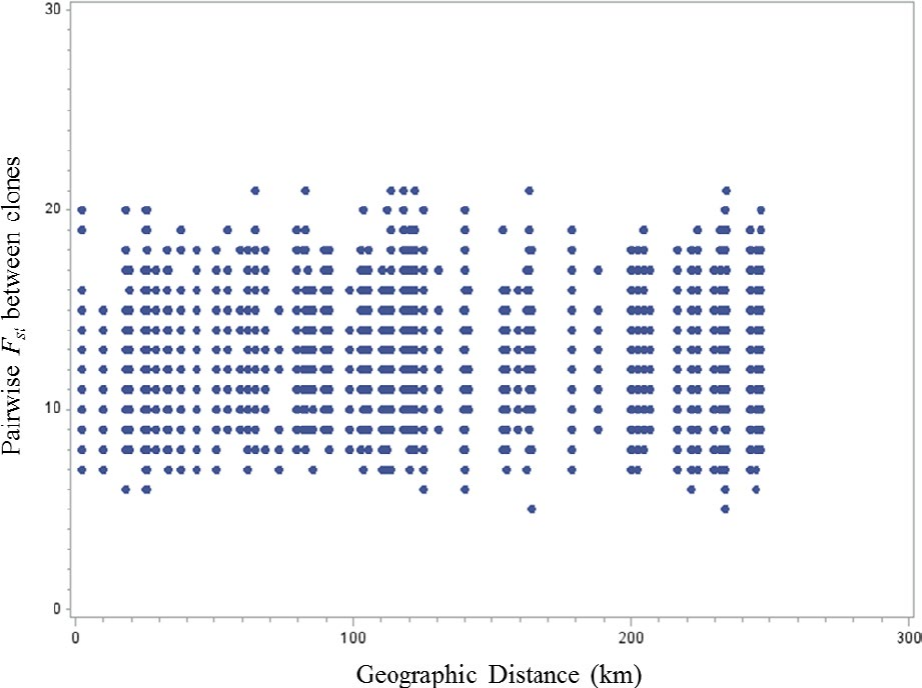
Genetic differentiation between clones (pairwise *F*_st_ values) plotted against their pairwise geographic distances

Principal coordinate analysis (PCoA) run on GenAlEx revealed no patterns among the 12 populations studied (Figure S1). Hence, we based our further genetic analysis on the four eco‐geographic regions to which the populations belong. A summary of the results from the genetic analysis is given in Table S1. The pairwise *F*_ST_ values among the clones pooled in the four eco‐geographic regions were small, but in general significant (Table 3). Largest *F*_st_ value (0.037) was found between the southwest and north eco‐geographic regions. However, population *F*_st_ values and their corresponding geographic distance were not correlated (*r* = .02; *p*‐value = .28) (Figure 4).

**Table 3:** Pairwise matrix of *F*_ST_ values for the four eco‐geographic regions used in the study (*F*_ST_ values below the diagonal; Probability *p* (rand ≥ data) based on 999 permutations is shown above diagonal)

Ecoregion	Central	North	SouthEast	SouthWest
Central	0.000	*0.017*	*0.224*	*0.001*
North	0.014	0.000	*0.007*	*0.001*
SouthEast	0.014	0.020	0.000	*0.005*
SouthWest	0.027	0.037	0.030	0.000

## 4 DISCUSSION

The 12 populations in the study were significantly differentiated with respect to spring phenology. Contrary to phenotypic data, populations were not substantially different to each other based on molecular analysis. The molecular and phenotypic data are in agreement regarding the poor relationship of genetic/phenotypic differentiation with isolation by distance. We interpret this as a reflection of the fragmentation in *T. cordata* distribution in Denmark (Fineschi et al., 2003; Lawesson, 2004) that could have led to low gene flow among the isolated populations but significant genetic drift within the small populations as predicted from theory (Hutchison & Templeton, 1999). The observed pattern of *Q*_ST_ > *F*_ST_ as well as the significant correlation between spring phenology and minimum temperatures in spring at the original population site indicate directional selection among *T. cordata* populations (Alberto et al., 2013; Leinonen et al., 2013; Whitlock & Guillaume, 2009), although the indications of low gene flow suggest that genetic drift can also have been important in shaping the present patterns in this trait (Hutchison & Templeton, 1999; Pickup, Field, Rowell, & Young, 2012). Conclusions are valid since our estimate of *Q*_ST_ for bud burst is downwards biased, but still much larger than *F*_ST_. Common gardens such as the clonal field trials in the present study helps in accounting for possible phenotypic plasticity exhibited by individual genotypes as they are grown in a single environment (Franks et al., 2014; Merilä & Hendry, 2014). However, environmental effects at the original population sites can still induce plastic/epigenetic effects (De Kort et al., 2014; Dewan et al., 2018; Groot, Wagemaker, Ouborg, Verhoeven, & Vergeer, 2018). Such epigenetic differentiation can be population specific and can be involved in adaptation to local environments (Bossdorf, Richards, & Pigliucci, 2008; Herrera & Bazaga, 2010). Hence, it is important to discern between the genetic and epigenetic regulation of adaptive traits among populations before drawing valid conclusions regarding mechanisms behind local adaptation (Grativol, Hemerly, & Ferreira, 2012; Richards, Bossdorf, & Verhoeven, 2010).

In our study, trees from warmer population origins flushed earlier at the test site (regression of population bud burst scores on minimum temperatures in spring at the population sites were significant). The populations grouped within each eco‐geographic region showed a clear pattern in their correlation between phenology and temperature in spring. This suggests that these populations are isolated by environment rather than distance (Sexton, Hangartner, & Hoffmann, 2013). In population genetics, it is often observed that the isolation by environment is more important for local adaptation than isolation by distance (Tiffin & Ross‐Ibarra, 2014); as under the influence of isolation by environment, it is likely that adaptive alleles undergo divergent selection within populations (Nadeau et al., 2016). Spatially divergent selection together with drift within isolated populations can cause the populations to be dissimilar phenotypically (high *Q*_ST_) even though there is little differentiation among populations at the molecular level (Savolainen et al., 2007). The occurrence of probable drift within these populations could result in reduced genetic variation within populations. Nevertheless, the broad sense heritability for spring phenology was found to be fairly high. Hence, a higher influence of divergent selection for this trait at individual population level is a reasonable explanation for the observed pattern of *Q*_ST_ > *F*_ST_ *,* even if underlying plastic responses induced by maternal environment effect among populations are present (De Kort et al., 2014). The ability to reproduce vegetatively (root/shoot suckers) (Koop, 1987a,b) helps in conserving the genetically distinct alleles within fragmented populations. The results suggest that scattered populations of *T. cordata* in Denmark, although with low gene flow, show a potential for fine scale adaptation through maintaining genetic diversity within populations in an adaptive trait such as spring phenology. Still, it remains a concern that the small and isolated populations can be particularly vulnerable to inbreeding depression and accumulation of deleterious mutant alleles, etc.

## 5 CONCLUSIONS

Our findings exemplifies that population differentiation in trees often occurs as a result of natural selection and neutral processes simultaneously. In our case, the populations have maintained a high level of genetic variation and thereby possess ability to respond to selection based on high level of heritability within populations together with high population differentiation. This suggests the presence of adaptive potential for spring phenology if exposed to strong selection. Plasticity also allows species to adapt to changes in growing conditions rapidly, and we cannot exclude that epigenetics have played an important role in creating the local adaptation (Verhoeven, vonHoldt, & Sork, 2016). Repeated assessments of budburst over years and ideally different climates will be required to compare the importance of genetic variation with phenotypic plasticity. The role of epigenetics is still poorly studied in woody species, but studies in Norway spruce (*Picea abies*) suggest that in at least some species the mechanism may be very efficient (Yakovlev et al., 2014). In the case of *Tiliaceae*, no studies have to our knowledge investigated the potential role of epigenetics in local adaptation, and this aspect therefore calls for more research in order to understand the adaptation mechanism in the species and support qualified discussions on the need for interventions as assisted migration or selection in the face of rapid climate change.

## CONFLICT OF INTEREST

None declared

## AUTHOR CONTRIBUTION

Albin Lobo was responsible for the design of study, analysis and interpretation of data, and writing of the manuscript. Ole Kim Hansen and Eva Ortvald Erichsen were responsible for the marker analysis, interpretation of molecular data, and writing of manuscript. Jon Kehlet Hansen was responsible for supervision of the work, writing and approving of manuscript. Birgitte Jacobsen was responsible for field data collection and statistical analysis of data. Erik Dahl Kjær was responsible for overall supervision of the study, conception and design of work, data interpretation, writing and approval of the manuscript.

## DATA ARCHIVING

Data for this study is available at University of Copenhagen—Electronic Research Data Archive (UCPH ERDA).
