# Supplementary figures and images for: Local adaptation through genetic differentiation in highly fragmented Tilia cordata populations
Source: Ecol Evol. 2018 May 7;8(12):5968–76. doi: 10.1002/ece3.4131 (PMC6024143; doi:10.1002/ece3.4131)

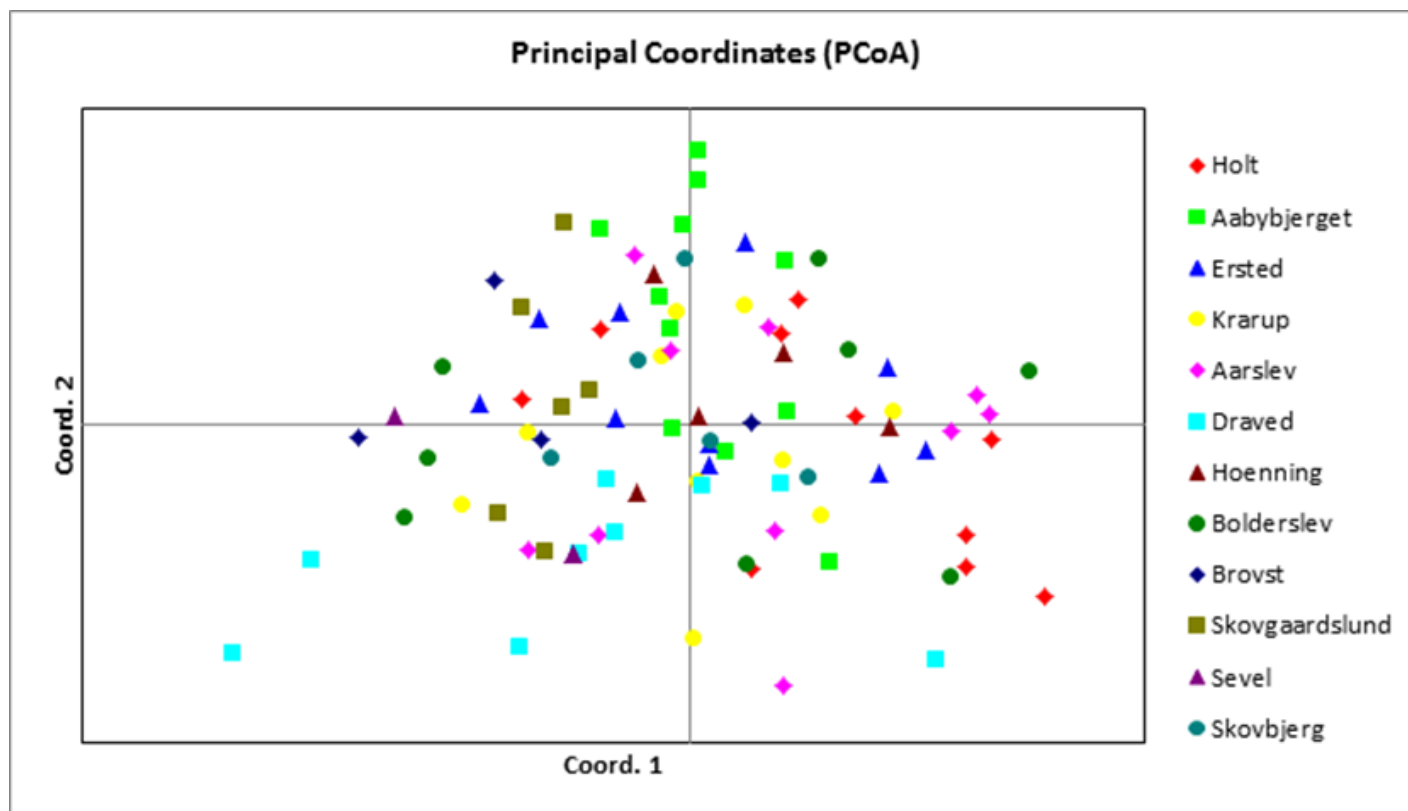

Supplement: Supplementary file 1 [file ECE3-8-5968-s001.pdf]
