# Supplementary material for: Local adaptation through genetic differentiation in highly fragmented Tilia cordata populations
Source: Ecol Evol. 2018 May 7;8(12):5968–76. doi: 10.1002/ece3.4131 (PMC6024143; doi:10.1002/ece3.4131)
Supplement: Supplementary file 2 [file ECE3-8-5968-s002.docx]

## **Supplementary material**

Table S1. Summary of genetic diversity measures for the 4 eco-geographic regions used in the study. Eco-geographic region means of diversity parameters with standard errors in parentheses

| **Eco-geographic region** | **N** | **Na** |  | **Ne** |  | **Na(rar)** | **Ho** |  | **He** |  | **F** |  |
| --- | --- | --- | --- | --- | --- | --- | --- | --- | --- | --- | --- | --- |
| Central | 28 | 8.11 | *(1.69)* | 4.91 | *(1.28)* | 6.76 | 0.64 | *(0.08)* | 0.66 | (0.09) | 0.01 | (0.03) |
| North | 31 | 8.11 | *(1.62)* | 4.55 | *(1.23)* | 6.59 | 0.62 | *(0.08)* | 0.62 | (0.09) | -0.03 | (0.03) |
| SouthEast | 18 | 7.00 | *(1.26)* | 4.19 | *(0.97)* | 6.51 | 0.64 | *(0.07)* | 0.65 | (0.07) | 0.00 | (0.03) |
| SouthWest | 14 | 5.78 | *(1.01)* | 3.40 | *(0.63)* | 5.78 | 0.60 | *(0.09)* | 0.60 | (0.08) | -0.01 | (0.05) |

*N number of sampled individuals, Na number of observed alleles, Ne effective number of alleles, Na(rar) allelic richness calculated via rarefaction, Ho observed heterozygosity, He expected heterozygosity, F fixation index*

Fig. S1: Principal Coordinate Analysis (PCoA) run on GenAlEx for the 12 populations
